# Cytoreductive Surgery plus Hyperthermic Intraperitoneal Chemotherapy Improves Survival for Patients with Peritoneal Carcinomatosis from Colorectal Cancer: A Phase II Study from a Chinese Center

**DOI:** 10.1371/journal.pone.0108509

**Published:** 2014-09-26

**Authors:** Chao-Qun Huang, Xiao-Jun Yang, Yang Yu, Hai-Tao Wu, Yang Liu, Yutaka Yonemura, Yan Li

**Affiliations:** 1 Department of Oncology, Zhongnan Hospital of Wuhan University, Hubei Cancer Clinical Study Center & Hubei Key Laboratory of Tumor Biological Behaviors, Wuhan, P.R. China; 2 NPO Organization to Support Peritoneal Dissemination Treatment, Osaka, Japan; H. Lee Moffitt Cancer Center & Research Institute, United States of America

## Abstract

**Background:**

Peritoneal carcinomatosis (PC) is a difficult clinical challenge in colorectal cancer (CRC) because conventional treatment modalities could not produce significant survival benefit, which highlights the acute need for new treatment strategies. Our previous case-control study demonstrated the potential survival advantage of cytoreductive surgery (CRS) plus hyperthermic intraperitoneal chemotherapy (HIPEC) over CRS alone. This phase II study was to further investigate the efficacy and adverse events of CRS+HIPEC for Chinese patients with CRC PC.

**Methods:**

A total of 60 consecutive CRC PC patients underwent 63 procedures consisting of CRS+HIPEC and postoperative chemotherapy, all by a designated team focusing on this combined treatment modality. All the clinico-pathological information was systematically integrated into a prospective database. The primary end point was disease-specific overall survival (OS), and the secondary end points were perioperative safety profiles.

**Results:**

By the most recent database update, the median follow-up was 29.9 (range 3.5–108.9) months. The peritoneal cancer index (PCI) ≤20 was in 47.0% of patients, complete cytoreductive surgery (CC0-1) was performed in 53.0% of patients. The median OS was 16.0 (95% confidence interval [CI] 12.2–19.8) months, and the 1-, 2-, 3-, and 5-year survival rates were 70.5%, 34.2%, 22.0% and 22.0%, respectively. Mortality and grades 3 to 5 morbidity rates in postoperative 30 days were 0.0% and 30.2%, respectively. Univariate analysis identified 3 parameters with significant effects on OS: PCI ≤20, CC0-1 and adjuvant chemotherapy over 6 cycles. On multivariate analysis, however, only CC0-1 and adjuvant chemotherapy ≥6 cycles were found to be independent factors for OS benefit.

**Discussion:**

CRS+HIPEC at a specialized treatment center could improve OS for selected CRC PC patients from China, with acceptable perioperative safety.

## Introduction

Peritoneal carcinomatosis (PC) from colorectal cancer (CRC) is characterized by the implantation of tumor nodules throughout the peritoneal cavity and production of intractable ascites. PC is found in about 8–15% CRC patients at first treatment [1], with a significant negative impacts on both the survival and the quality of life because of refractory ascites, progressive intestinal obstruction and uncontrollable abdominal pain, with approximately 30% of the CRC patients died from this problem [2]. Up to now, the oncology community usually considers CRC PC as a virtually untreatable öterminal condition, for which only palliative measures such ?as systemic chemotherapy, with or without limited surgery and best support care, with limited median overall survival (OS) about 6 months [3]–[5].

Increasing studies on this problem has gradually resulted in revolutions in both the basic pathological sciences and clinical approaches to CRC PC. Different from CRC liver metastases, CRC PC is now regarded as regional tumor progression, suitable for radical therapeutic strategies with cytoreductive surgery (CRS) and hyperthermic intraperitoneal chemotheropy (HIPEC), which are likely to achieve prominent clinical benefit in selected patients [6]–[8]. Although only one phase III prospective randomized controlled clinical trial [6] demonstrated the superiority of this new strategy, it has been considered to justify this comprehensive treatment, much like the evolutionary history of liver resection as a standard procedure for selected patients with CRC liver metastasis. Nevertheless, overwhelming majority of relevant researches came from the Western countries, and there have been no systematic clinical studies from China, where the problem is particularly acute due to the large number of such patients. To address the clinical problem, we have performed a series of preclinical and clinical studies on the feasibility, efficacy and safety of this multidisciplinary therapeutic approach in animal models [9] and in clinical setting [10]–[12], and established a designated CRS+HIPEC program at our institution, which has been in steady operation for over 10 years. As our phase I study [10] and case-control study [12] (the OS was 8.5 in Control group versus 13.7 months in Study group, *P* = 0.02) suggested potential therapeutic benefit of CRS+HIPEC for CRC PC patients, we proceeded to this prospective phase II study with enlarged sample size to further investigate the efficacy and safety of CRS+HIPEC and postoperative chemotherapy for the treatment of CRC PC in Chinese patients.

## Patients and Methods

### Ethics statement

All patients had signed the informed consent form, and the study protocol was approved by the institutional review board of Zhongnan Hospital of Wuhan University. This study was registered in the clinical trial registry by US National Cancer Institute. The registry name is “Surgery plus Intraoperative Peritoneal Hyperthermic Chemotherapy (IPHC) to Treat Peritoneal Carcinomatosis” and ClinicalTrials.gov Identifier is “NCT00454519”. Detailed study information is available at Clinical-Trials.gov (http://www.clinicaltrials.gov/ct2/show/NCT00454519).

### Patients Selection

**Figure 1:**
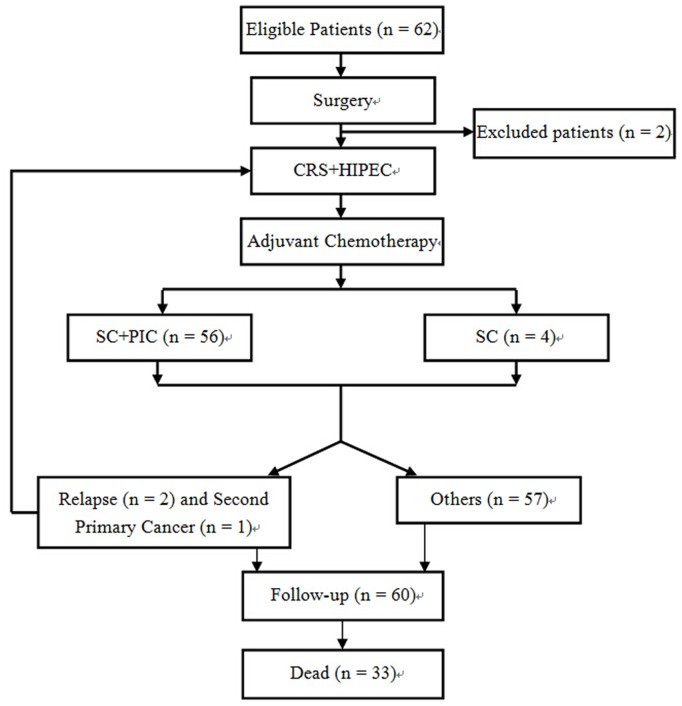
The flowchart of this phase II clinical study. Initially 62 patients were enrolled into this study, and 2 were excluded. A total of 60 patients underwent 63 CRS+HIPEC procedures. CRS = cytoreductive surgery, HIPEC = hyperthermic intraperitoneal chemotherapy, SC = systemic chemotherapy, PIC = perioperative intraperitoneal chemotherapy.

This prospective phase II study included 60 consecutive Chinese patients of CRC PC treated by 63 CRS+HIPEC procedures from February 2005 to October 2013 at the Department of Oncology, Zhongnan Hospital of Wuhan University. The preoperative evaluations, major inclusion and exclusion criteria were reported previously [11]. In addition, those patients were excluded who received neoadjuvant chemotherapy or any adjuvant chemotherapy within preoperative 6 months. One patient with advanced age and another one with extensive CRS but could not tolerate HIPEC were excluded. The flowchart of this study was shown in Figure 1.

### CRS plus HIPEC Procedure

CRS+HIPEC were conducted by a designated team focusing on PC treatment, with detailed description reported previously [10]–[12]. In brief, maximal CRS was performed to remove the primary tumor with acceptable margins, any involved adjacent tissues and organs, regional lymph nodes, and peritonectomy [13]. Unresectable tumors were cauterized with ball-tipped electrosurgical device at the maximal electric power, especially on the edge of tumor nodules. The completeness of cytoreduction (CC score) [13] was evaluated before HIPEC, which was implemented by the open Colliseum technique, with 120 mg of cisplatin and 30 mg of mitomycin C each dissolved 6 L of heated saline (drug concentration cisplatin 20 µg/mL, mitomycin C 5 µg/mL), as these concentration has been confirmed to be safe and effective for HIPEC by Fujimoto et al [14]. The heated perfusion solution was infused into the peritoneal cavity at a rate of 500 mL/min through the inflow tube introduced from an automatic hyperthermia chemotherapy perfusion device. The temperature of the perfusion solution in peritoneal space was kept at 43.0±0.5 °C and monitored with a thermometer on real time. The total HIPEC time was 90 min, after which the perfusion solution in the abdominal cavity was removed through the suction tube. After operation, the patient was delivered to the intensive care unit for recovery.

### Postoperative Chemotherapy

Adjuvant chemotherapy included systemic chemotherapy mainly with FOLFOX (oxaliplatin, leucovorin and 5-FU) or FOLFIRI (irinotecan, leucovorin and 5-FU) regimens, and perioperative intraperitoneal chemotherapy (PIC) through the intraperitoneal chemotherapy port mainly using docetaxel (75 mg/m^2^, on day 1, every 3 weeks) and carboplatin (at Calvert formula: area under the curve, AUC 5; on day 1, every 3 weeks), all dosed on the base of body surface area calculation [7], [15], [16]. PIC were launched after the patients postoperative physical conditions recovered, and systemic chemotherapy were delivered with PIC synchronously or alternately.

### Study Endpoints and Their Definition

The primary endpoint was the disease specific overall survival (OS), defined as the time interval from the first treatment to death due to the disease for synchronous PC, and from CRS+HIPEC to death due to the disease for metachronous PC. The secondary endpoints were the perioperative adverse events, defined as complications directly attributable to the treatment within 30 days of CRS+HIPEC, based on NCI Common Terminology Criteria (CTC) for Adverse Events version 4.0 [17].

### Follow-up

All patients received regular follow-up once every 3 months for the first 2 years, and once every 6 months thereafter. The follow-up package included physical examination, serum tumor markers (CEA/CA125/CA19-9), abdominopelvic CT scans and/or gastrointestinal iodine contrast media. The last follow-up was on March 11, 2014, and the overall follow-up rate was 100%.

### Statistical Analysis

All data analyses were performed using the SPSS statistical software for windows, version 20.0. The numerical data were directly recorded, and the category data were recorded into different categories. OS comparisons were analyzed with Kaplan-Meier cumulative survival curve and log rank test, and multivariate Cox regression analysis was performed to delineate the independent predictors. The OS comparisons were further stratified by PCI scores, with PCI ≤20 defined as low PCI (LPCI), and >20 as high PCI (HPCI) [13]. A 2-sided *P*<0.05 value was considered as statistically significant.

## Results

### Major Clinicopathological Characteristics and Perioperative Treatment

A total of 60 CRC PC patients received 63 CRS+HIPEC procedures, including 3 patients each receiving 2 CRS+HIPEC procedures (2 patients due to tumor recurrence and 1 patient due to the second primary cancer). Major clinico-pathologic characteristics of the patients were listed in Table 1.

**Table 1:** Major clinico-pathologic characteristics of the 60 patients^a.^

Items	Value, n (%)	
Gender		
Male	26 (43)	
Female	34 (57)	
Age (years)		
<60	46 (77)	
≥60	14 (23)	
Median KPS score (range)	80 (50–100)	
Primary tumor		
Carcinoma of colon	35 (58)	
Carcinoma of rectum	25 (42)	
Histopathology		
Adenocarcinoma, well/intermediately differentiated	26 (43)	
Adenocarcinoma, poorly/mucinous/signet-ring cell carcinoma	34 (57)	
PC timing b		
Synchronous	24 (40)	
Metachronous	36 (60)	
PCI scores b		
≤20	28 (47)	
>20	32 (53)	
Median PCI scores (range)	21 (1–39)	
Ascites at surgery a		
≤1,000 mL	45 (71)	
>1,000 mL	18 (29)	
Surgical procedures-organ resection		
Resection of jejunum	3 (5)	
Resection of ileum	16 (29)	
Resection of ileocecus	16 (29)	
Ascending colectomy	21 (38)	
Transverse colectomy	26 (47)	
Descending colectomy	10 (18)	
Sigmoidectomy	14 (25)	
Rectectomy	14 (25)	
Splenectomy	4 (7)	
Resection ovarian/fallopian tube	20 (36)	
Hysterectomy	14 (25)	
Partial hepatectomy	2 (4)	
Cholecystectomy	6 (11)	
Number of organ resected b ^,^ c		
1–3 resections	33 (59)	
4–7 resections	23 (41)	
Peritonectomy b		
Greater/Lesser/Omentum	60 (100)	
Left diaphragmatic copula	15 (25)	
Right diaphragmatic copula	21 (35)	
Right colon gutter	20 (33)	
Left colon gutter	17 (28)	
Liver round ligament/sickle ligament	19 (32)	
Douglas pouch	5 (8)	
Anterior wall peritoneum	20 (33)	
Pelvic peritoneum	32 (53)	
Mesenteric fulguration	35 (58)	
Peritoneal resection area b		
1–3 resections	30 (50)	
4–6 resections	15 (25)	
7–12 resections	15 (25)	
CC scores b		
0	17 (28)	
1	15 (25)	
2–3	28 (47)	
Number of anastomosis b		
None or Ostomy only	26 (43)	
= 1	27 (45)	
>1	7 (12)	
Postoperative chemotherapy cycles		
<6	15 (15)	
≥6	45 (75)	
Median postoperative chemotherapy cycles (range)	8 (2–18)	
Postoperative intraperitoneal chemotherapy cycles d	4 (1–10)	
Median duration of hospitalization (days) (range)	21 (11–58)	
Median follow-up (months)(range)	29.9 (4.5–109.9)	

a 3 patients each underwent 2 operations;

b According to the first surgery;

c 4 patients without organ resection;

d Except for 4 patients who developed intestinal fistula after operation.

Surgical procedures and major intraoperative parameters including blood loss, duration of operation, and fluid balance parameters were listed in Table S1.

After operation, all the 60 patients received systemic chemotherapy and 56 patients received PIC procedures except four patients who had intestinal leakage. The PIC was delivered on day 8 post-operation (range, 3–14 days), except one patient who received PIC on postoperative day 20 due to severe gastroplegia, and with median 4 cycles of PIC for each patient. The median cycles of adjuvant chemotherapy (SC and/or PIC) was 8 (range, 2–18). None of the patients received any molecular targeting agents.

## Survival Analysis

**Figure 2:**
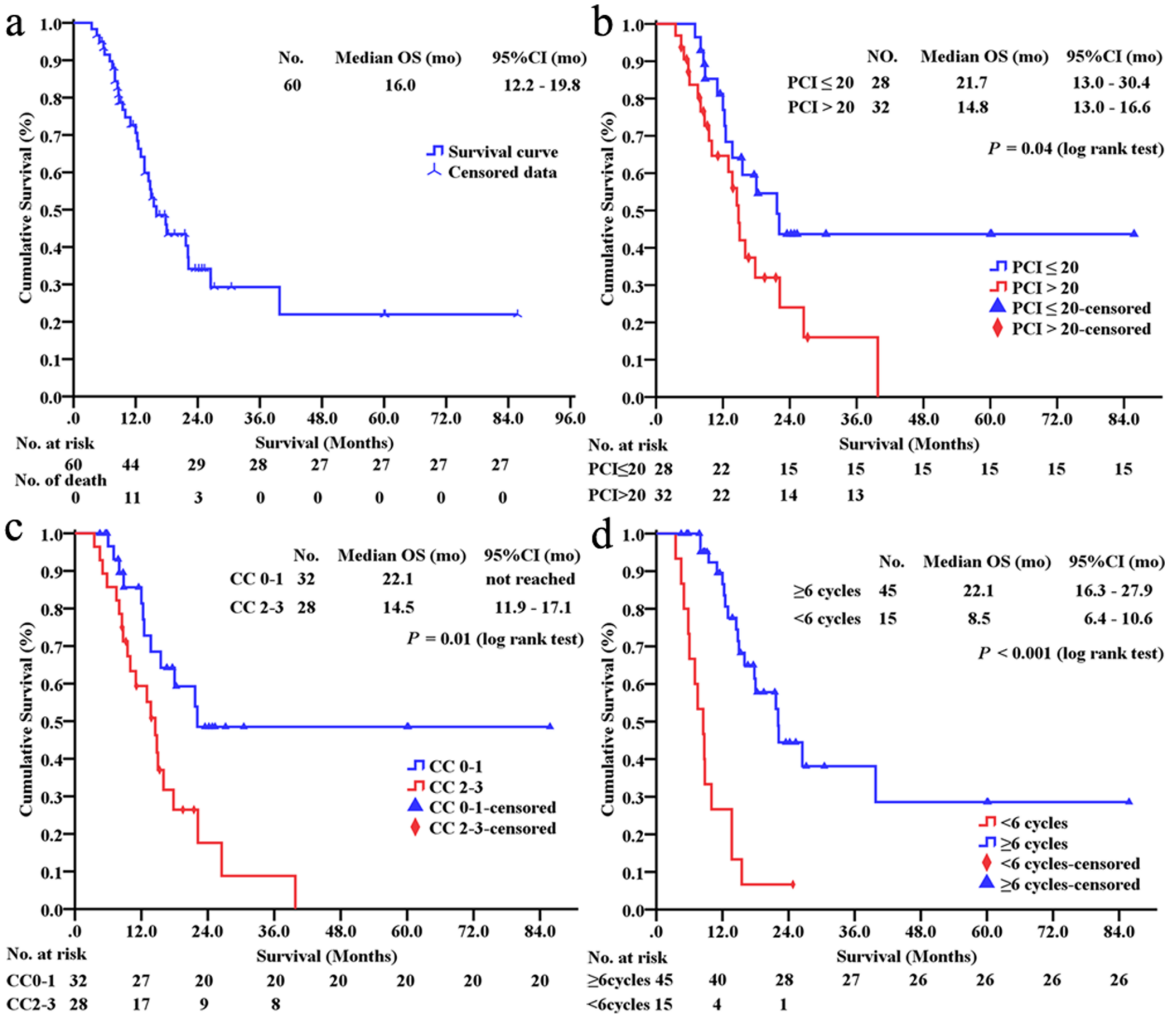
Kaplan-Meier curves. The disease-specific overall survival in patients with CRC PC treated by CRS+HIPEC and adjuvant chemotherapy regimen (**a**). The statistical significance in overall survival (OS) comparisons of those patients including PCI (**b**), CC (**c**) and postoperative adjuvant chemotherapy cycles (**d**). mo = months, CRC = colorectal cancer, PC = peritoneal carcinomatosis, CRS = cytoreductive surgery, HIPEC = perioperative intraperitoneal chemotherapy, PCI = peritoneal cancer index, CC = completeness of cytoreduction.

At the last follow-up, the median OS was 16.0 (95% CI 12.2–19.8) months (Figure. 2a). The 1-, 2-, 3- and 5-year survival rates were 70.5%, 34.2%, 22.0% and 22.0%, respectively. The OS comparisons were stratified based on major clinico-pathological factors (Table S2.). Male patients, colon cancer PC, well/intermediately differentiated adenocarcinoma, synchronous PC and the volume of ascites at surgery ≤1,000 mL had a tendency for improved OS; and PCI ≤20 (*P* = 0.04, Figure. 2b) and CC0-1 (*P* = 0.01, Figure. 2c) and postoperative chemotherapy cycles ≥6 (*P*<0.001, Figure. 2d) could obtain statistically better OS benefit.

## Special Analysis on Long-term Survivors

There were 15 patients surviving over 20 months (Table 2), including 7 patients who had disease free survival (DFS) with LPCI and CC-0 resection, 3 patients survived with tumor (SWT), and 5 patients died with HPCI and non-CC-0 resection. It worth noting that 4 patients with HPCI and CC-2/3 resection also achieved long-term OS of 21.5, 22.2, 26.5 and 39.8 months, respectively.

**Table 2:** Major treatment and follow-up features of long-term survivors (OS >20 months).

No.	Gender/age(yr)	PC origin/PC timing	PCI	Histopathology	CRS	CC	Survival(months)	Comments
1	M/36	Colon ca,Synchronous PC	6	Adenocarcinoma, well differentiated	Left hemicolectomy, greater omentum resection, left peritoneum and musculus trasversus abdominis, mesenteric fulguration	0	85.8, DFS	
2	M/36	Colon ca,Synchronous PC	First:15Second: 0	Adenocarcinoma, intermediatelydifferentiated	The first surgery: transverse colectomy, resection of part jejunum, greater omentum resection;The second surgery: rectectomy, colostomy of sigmoid colon (second primary cancer: rectal cancer) without HIPEC	0	First:55.0DFSTotal: 60.2, DFS	SAE (the first surgery): abdominal hemorrhage 4 h post operation, reoperation to stop bleeding; Pelvic and abdominal adhesions were found in second surgery
3	M/47	Colon ca,Synchronous PC	15	Adenocarcinoma, intermediatelydifferentiated	Ascending colectomy, resection of ileocecus	0	60.0, DFS	
4	F/57	Colon ca,Synchronous PC	24	Adenocarcinoma, intermediatelydifferentiated	Right hemicolectomy, hysterectomy and resection ovarian/fallopian tube, greater omentum, left and right diaphragmatic copula peritoneum, left and colon gutter peritoneum, pelvic peritoneum, liver round ligament, mesenteric fulguration	0	27.2, SWT	
5	F/37	Colon ca,Metachronous PC	1	Adenocarcinoma, mucinous	Greater omentum	0	25.3, DFS	
6	F/65	Colon ca,Metachronous PC	5	Adenocarcinoma, intermediatelydifferentiated	Resection ovarian/fallopian tube, greater omentum, liver round ligament	0	24.8, DFS	
7	F/68	Colon ca,Synchronous PC	6	Adenocarcinoma, intermediatelydifferentiated	Right hemicolectomy, resection ovarian/fallopian tube, greater/lesser omentum	0	24.5, DFS	
8	M/60	Colon ca,Metachronous PC	First:15Second: 18	Adenocarcinoma, mucinous	The first surgery: right hemicolectomy, greater omentum, pelvic peritoneum resection, mesenteric fulgurationThe second surgery: resection of ileocecum, rectectomy; received HIPEC as before	First:1Second: 2	30.5, SWT	Not found vast pelvic and abdominal conglutination in second surgery
9	F/55	Colon ca,Metachronous PC	16	Adenocarcinoma, intermediatelydifferentiated	Left hemicolectomy, rectectomy, hysterectomy and resection ovarian/fallopian tube, greater omentum	0	24.2, DFS	
10	M/41	Rectal ca,Metachronous PC	20	Adenocarcinoma, poorlydifferentiated	Rectectomy, greater omentum, left/right diaphragmatic copula resection, colon sigmoideum colostomy	1	22.1, D	
11	F/54	Colon ca,Metachronous PC	7	Adenocarcinoma, mucinous	Sigmoidectomy, rectectomy, greater omentum, pelvic peritoneum resection	1	21.7, D	
12	M/30	Colon ca,Synchronous PC	First: 32Second: 39	Adenocarcinoma, mucinous	The first surgery: right hemicolectomy, resection of part jejunum, greater omentum, left diaphragmatic copula, left/right colon gutter, liver round ligament/sickle ligament resection, mesenteric fulgurationThe second surgery: lump-like tumor on hepatic portal, subcutaneous plate-like tumor; without HIPEC	First:2Second: 3	39.8, D	
13	F/37	Colon ca,Metachronous PC	26	Adenocarcinoma, mucinous	Descending colectomy, resection of part jejunum, left/right diaphragmatic copula, left/right colon gutter, anterior wall peritoneum, pelvic Peritoneum resection, mesenteric fulguration	2	26.5, D	
14	M/26	Colon ca,Synchronous PC	28	Adenocarcinoma, mucinous	Greater/lesser omentum, liver round ligament/sickle ligament, anterior wall peritoneum resection, mesenteric fulguration	2	22.2, D	
15	M/43	Colon ca,Synchronous PC	39	Adenocarcinoma, mucinous	Pancolectomy, greater omentum	3	21.5, SWT	

M = male, F = female, ca = carcinomatosis, D = died, DFS = disease free survival, SWT = survival with tumor, SAE = serious adverse event.

## Serious Adverse Events (SAE)

SAE (grade 3–5) occurred in 19 (30.2%) of 63 procedures, including postoperative intestinal obstruction (n = 10), intestinal leakage (n = 4), hemorrhage (n = 1), diarrhea (n = 1), septicaemia (n = 1), severe hypoalbuminemia (n = 1), and delirium (n = 1). Detailed accounts of the 19 SAEs were listed in Table 3; No SAE-related death occurred in perioperative period.

**Table 3:** Detail data of the patients with SAE # (n = 19, 30.2%).

Events	Gender/Age	Primary tumor	PC time	PCI	Resection		No.Anastomosis	CC	Surgery time (min)	POD	SAEs	Intervention a	OS b(month)
					Organ	Peritoneal							
Intestinal obstruction (n = 10, 15.9%)													
	M/27	Colon	Syn	24	5	2	1	3	485	4	CIO/AI	CT	8.0, D
	M/47^c^	Colon	Met	27	1	1	1	2	225	7	IIO/AI	CT	4.5, D
	M/52	Colon	Met	10	4	3	1	0	610	13	CIO/AI	CT	8.0, D
	M/47	Colon	Syn	26	5	2	1	1	670	13	CIO/AI	CT	15.0, D
	M/25	Colon	Syn	28	1	6	1	2	650	12	CIO/AI	CT	22.2, D
	F/62	Colon	Met	18	1	5	1	3	510	8	IIO/AI	CT	15.3, S
	F/72	Rectum	Met	5	3	2	1	0	405	6	CIO/AI	CT	8.8, S
	M/61	Colon	Syn	29	1	9	1	3	585	7	IIO/AI	CT	11.1, S
	F/45	Colon	Met	24	2	5	2	1	390	5	IIO/AI	CT	5.8, S
	F/31	Colon	Met	24	5	6	2	1	740	5	CIO/AI and combined gastroplegia	CT	5.5, S
Intestinal leakage (n = 4, 12.1%)													
	M/33	Colon	Met	26	4	6	1	2	540	16	limited peritonitis syndrome	CT	5.0, D
	M/47^c^	Colon	Met	27	1	1	1	2	610	7	sepsis, generalized peritonitis, abdominal abscess	CT	NA
	F/55	Colon	Syn	16	7	1	1	0	525	6	limited peritonitis syndrome	reoperation	24.2, S
	F/71	Rectum	Met	9	1	1	1	0	300	4	limited peritonitis syndrome	CT	7.0, S
Hemorrhage (n = 1, 1.6%)													
	F/36	Rectum	Syn	15	0	1	2	0	210	4h	Hemorrhage shock	reoperation	60.2, S
Diarrhea (n = 1, 1.6%)													
	M/60^c^	Colon	Met	26	1	4	1	2	465	8	over 8 stools per day and abdominal pain	CT	13.7, D
Septicemia (n = 1, 1.6%)													
	M/60^c^	Colon	Met	26	1	4	1	2	465	8	*Staphylococcus aureus* septicemia	CT	NA
Hypoalbuminemia (n = 1, 1.6%)													
	M/47	Colon	Met	15	3	2	1	1	420	2	16.1 g/L	CT	11.5, S
Delirium (n = 1, 1.6%)													
	M/60^c^	Colon	Met	26	1	4	1	2	465	8	grades 3	drug intervene	NA
Median	47			24	2	3	1	2	485	7			

a all patients recovered after intervention;

b none of the patients died of SAE;

c different SAEs developed in the same patient;

# Common Terminology Criteria for Adverse Events version 4.0;

POD = on postoperative days, M = male, F = female, Syn = synchronous, Met = metachronous, CIO = complete intestinal obstruction, IIO = incomplete intestinal.

obstruction, AI = adynamic ileus CT = conservative treatment D = death, S = survival, NA = not applicable.

Ten patients developed gastrointestinal obstruction within 2 weeks after operation, of whom 9 patients gradually recovered by active conservative therapy, and 1 patient with severe gastroplegia restored to normal gastrointestinal function 13 days after operation; there were no obvious electrolyte disturbance, serious infection or sepsis during the conservative therapy. Four patients developed intestinal leakage, including 2 patients with mild anastomosis leakage and limited peritonitis syndrome on postoperative days 4 and 16, respectively, and these 2 patients recovered after 3 months and 7 days, respectively. The third patient developed colonic stump fistula and vaginal-stump fistula on postoperative days 6, received a reoperation to reconstruct the anastomosis and repair vaginal-stump on postoperative days 33, and the patient is surviving and well with DFS of 24.2 months. The fourth patient developed severe anastomosis leakage, generalized peritonitis, abdominal abscess formation and septicemia due to *Escherichia coli*. With abdominal drainage, intensified antibiotics, and total parenteral alimentation nutrition support, this patient survived 3 months after the procedure. One patient with abdominal hemorrhage 4 h post operation had reoperation to stop the bleeding. This patient recovered well and he had DFS of 55.0 months, until November 2013 when he developed a second primary rectal cancer, which was successfully resected, and he is living well with no evidence for any tumor recurrence, with a total DFS of 60.2 months. Another SAE case was a 60-year-old male patient who developed septicemia along with grade 3 inflammatory diarrhea, abdominal pain and delirium on day 8 post-operation, due to infection by *Staphylococcus aureus* as confirmed by blood culture. The septicemia was controlled in 5 days after antibiotics therapy, and the patient fully recovered in about 10 days. The patient survived for 13 months. The last SAE case developed sustained grade 3 hypoalbuminemia (16.1 g/L) from postoperative days 2 to 9, and treated with high-volume plasma and albumin transfusion, and the patient gradually recovered without serious consequences.

A binary logistic regression analysis was conducted to study the correlation of SAEs with major treatment parameters. No significant correlations between SAEs and gender, age, KPS scores, primary tumor, PC time, histopathology, organ and peritoneal resection area, number of anastomosis, operation duration, PCI scores, and CC scores.

Other adverse events (grades 1–2) occurred in 54 (85.7%) of the 63 procedures, including mild hypoalbuminemia (n = 36), liver and kidney dysfunctions (n = 12), delayed incision healing (n = 4), atelectasis (n = 1), and deep vein thrombosis (n = 1).

## Univariate and Multivariate Analysis on Predictors of Survival

Ten parameters were included for univariate analysis respectively (Table 4), and 3 covariates indicative of improved survival including PCI ≤20, CC0-1, and postoperative chemotherapy cycles ≥6.

**Table 4:** Analysis of independent factors influencing survival.

Covariate	Univariate analysis				Multivariate analysis			
	χ^2^	*P*	HR	95% CI	χ^2^	*P*	HR	95% CI
PCC (≥6 *vs.*<6)	22.20	**<0.001**	5.8	2.8–12.0	24.91	**<0.001**	7.2	3.3–15.5
CC score (CC0-1 *vs.* CC2-3)	7.58	**0.01**	2.8	1.3–5.6	10.26	**<0.001**	3.4	1.6–7.2
PCI score (≤20 *vs.* >20)	4.03	**0.045**	2.1	1.0–4.2				
Primary tumor (colon *vs.* rectum)	2.78	0.10	1.9	0.9–3.9				
PC timing (Syn *vs.* Met)	2.71	0.10	1.8	0.9–3.8				
Histopathology (well *vs.* poorly differentiated)	2.36	0.12	1.8	0.8–3.9				
Age (≥60 *vs.* <60)	1.81	0.18	2.1	0.7–5.9				
Gender (male *vs.* female)	1.43	0.23	1.5	0.3–3.1				
Ascites (≤1,000 mL *vs*. >1,000 mL)	1.25	0.26	1.5	0.7–3.2				
SAE (no *vs.* yes)	0.00	0.99	1.0	0.4–2.3				

PCC = postoperative chemotherapy cycles, Syn = synchronous, Met = metachronous.

Multivariate Cox regression analysis identified 2 variables including CC scores and postoperative adjuvant chemotherapy cycles as independent predictors for better survival (Table 4). Compared with CC2-3 and postoperative chemotherapy cycles <6, CC0-1 and postoperative chemotherapy cycles ≥6 were about 3 times (Hazard Ratio = 3.4, 95% CI 1.6–7.2, *P*<0.001) and 7 times (Hazard Ratio = 7.2, 95% CI 3.3–15.5, *P*<0.001) more likely to improve survival, respectively.

## Analysis of Predictors of Survival for HPCI Patients: Univariate and Multivariate Analysis

As our data contained 32 (53%) cases with HPCI (PCI >20), we conducted a subgroup analysis on such patients. Survival analysis of the patients with HPCI by Kaplan-Meier (Table S3.) found the median OS was 15.0 *vs.* 9.5 months in synchronous (n = 15) *vs.* metachronous (n = 17) subgroups (*P* = 0.12); 17.8 *vs.* 6.0 months in postoperative chemotherapy cycles ≥6 (n = 23) *vs.* <6 (n = 9) subgroups (*P*<0.001); 14.8 *vs*. 13.7 months in no-SAE (n = 23) *vs.* SAE (n = 13.7) subgroups (*P* = 0.21), respectively.

Four parameters were included for multivariate analysis, and only postoperative adjuvant chemotherapy cycle was identified as independent predictor for better survival. The chemotherapy over 6 cycles was about 15 times more likely to improve survival in contrast to less 6 cycles (Chi-square = 15.0, 95% CI 3.7–61.0, *P*<0.001).

## Discussion

Over the past 10 years, we have established a designated PC program, with both preclinical [9], [18] and clinical studies [10], [11] to demonstrated the efficacy of CRS+HIPEC for PC treatment. This phase II clinical study is a part of our comprehensive treatment strategy for PC, producing several lines of new evidence. First, for CRC patients with both synchronous and metachronous PC, the median disease specific OS could reach 16.0 months (95% CI 12.2–19.8 months) by such combined treatment approach, and the 3-yr survival rate could reach 22.0%, even though none of them received any molecular targeting therapy. Second, the median disease specific OS could reach 22.0 months for patients with synchronous PC, but 14.5 months for those with metachronous PC. Although such differences did not reach statistical significance, possibly due to follow-up durations and sample size, they do suggest that CRC patients with synchronous PC could obtain better survival benefit from such treatment. Third, completeness of CRS has an important independent impact on survival. Therefore, every effort should be made during surgery to reduce the tumor burden. Fourth, adjuvant systemic chemotherapy after CRS+HIPEC could bring about significant survival advantages for such patients. All the 15 long-terms survivors had over 6 cycles of post-operative systemic chemotherapy. These major results, once again, provide data to support the rational for CRS+HIPEC, that is to remove the bulky tumor by surgical resection, to eliminate the micro-metastasis and seeding nodules by the heated chemotherapeutic perfusion, and to boost the treatment efficacy by postoperative systemic and intraperitoneal chemotherapies [6], [19].

To our knowledge, there have been about 15 clinical trials (Table S4.) on the treatment of CRC PC, including 5 phase I studies [20]–[24], 9 phase II studies [25]–[33], and 1 phase III study [6]. These studies covered the surgical procedures, drug treatment selection, pharmacological evaluation and adverse events. In 5 phase I studies on a total number of 109 patients, it was found that HIPEC at temperature 40–46°C for 30–90 min, with oxliplatin, MMC, pegylated liposomal doxorubicin, and irrinotecan, were feasible with acceptable efficacy and side effects. In 9 phase II studies on 562 CRC PC patients, the median overall survivals ranged from 19.8 months to 41.0 months, and 3-yr survival rate reaching 63.7% at the most. In the only phase III study, the median OS was 22.2 months in CRS+HIPEC *vs.* 12.6 months in control group (Surgery+Systemic Chemotherapy). Moreover, all these studies also indicated that while the treated patients obtained better efficacy the SAEs were also acceptable. Therefore, the CRS+HIPEC have become increasingly accepted as the treatment of choice at designated centers for selected patients with CRC PC. Moreover, a nationwide application of standardized CRS+HIPEC protocol in the Netherlands has produced more encouraging long-term survival of about 33.0 months [34]. In addition, 3 large sample-size multicenter retrospective studies in France and Italy also showed a mean OS of 23.4 months [7], [16], [35]. Compared with these international studies, our results on Chinese patients treated with standardized CRS+HIPEC protocols have added, for the first time, the new evidence from China on this increasing international database.

Several factors have been recognized to have important impacts on the clinical outcomes of this combined treatment, including PCI, CC, SAEs, lymph nodes status, synchronous vs. metachronous PC, and systemic chemotherapy. For PCI, the Sugarbaker PCI system has been adopted as the current standard recording system [13], and out of the total 39 cores, PCI ≤20 is usually regarded as LPCI and >20 is regarded as HPCI [36], [37]. In this study, of the 32 patients (53% of the total) with HPCI, the median OS was 14.8 months (95% CI 13.0–16.6 months). This is comparable to most other studies [35], [38], [39] showing a median OS of about 12 months for patients with PCI >20. Sugarbaker et al [38]. also reported the 5-year survival rates of 50%, 20%, and 0%, respectively for patients with PCI ≤10, 11–20, and >20. Our results suggest that HPCI Chinese patients still could benefit from HIPEC procedure rather than the traditional treatment, at the specialized treatment center. However, it was indeed the one cause to shorten OS in this study in contrast to LPCI patients. Therefore, for HPCI patients especially for patients with synchronous PC, intensified chemotherapy to reduce the tumor burden first and then followed by CRS+HIPEC could be a sensible strategy in future work.

For CC score, CC0 patients had much better survival benefit than CC1-3 patients, with reported median OS 33.0 months *vs.* 10.0 months for CC0 *vs.* CC1-3 [6], . In this study, 17 patients with CC0 cytoreduction reached mean survival of 50.1 months, again supporting the effort to reduce tumor burden as much as possible. Among these 17 patients with a CC0 resection, 15 were in LPCI category and 2 in HPCI category, again confirming the importance of selecting LPCI patients for treatment. To achieve complete cytoreduction, however, a wide resection is usually necessary involving several organs and abdominal regions. In our study, usually 2 to 3 organ resections and 3 to 4 peritoneal regions are resected in one CRS procedure, which is long, complex and technically difficult. This will inevitably increase the blood and fluid losses, hemodynamic disturbances, and increase risks for SAEs [40], with perioperative morbidity rate ranging from 14.8% to 57.0% and mortality rate from 0.0% to 12.0% [8]. In 2 multicenter studies by Glehen et al [7] and Elias et al [16], the perioperative mortality rate was 4% and 3%, respectively. In the present study, the 30-day perioperative SAE rate of 30.2% and mortality rate of 0% also fell into the reported ranges. The overall morbidity and mortality are comparable with conventional gastrointestinal surgery and acceptable, if patients are treated at specialized PC centers, as demonstrated in a meta-analysis by Chua et al [41]. It was reported in a recent Japanese study that HPCI and complete resection rates were closely associated with high morbidity rate [42]. In our study, however, our binary logistic regression did not reveal any significant correlation between SAE and HIPEC with CC0-1. Although SAE was not identified as an independent prognostic factor, possibly due to relatively small sample size, it has significant negative impacts on the quality of life, and increase the treatment cost. Therefore, intensified perioperative care should be delivered so as to minimize the risks for SAEs.

Our study found that postoperative adjuvant chemotherapy also played an important role in the comprehensive therapeutic strategy. All the 60 patients received systematic chemotherapy and 56 cases had PIC within postoperative 4 weeks. Because of peritoneal-plasma partition [43], only a minimal chemotherapy drugs could penetrate into the abdominal cavity, resulting in reduced drug concentration in PC nodules. Appling PIC in addition to systematic chemotherapy could have double effects on PC nodules from a bi-directional approach, which has been proved to play a key role for better survival in recent reports [16], [25], [28], [29], and in our multivariate analysis of predictors of survival.

Of particular attention is the 15 patients with prolonged survival over 20 months in this series, including 5 patients survived over 30 months and 1 over 80 months. Five major factors for such favorable outcome are synchronous PC, well/intermediately differentiated adenocarcinoma, mucinous adenocarcinoma, LPCI and CC0-1 resection. Seven of the 15 patients with LPCI and CC0 showed the median OS from 24.2 months to 85.8 months in this study, again confirming the crucial importance of complete cytoreduction for better survival, as reported previously [6], [29], [34]–[36], [44]–[48]. However, what deserve special attention are four patients with HPCI and CC2-3 resection still obtained long-term survival (OS ranging from 21.5–39.8 months). These patients shared the following features in common: less than 45 years old, synchronous PC, mucinous adenocarcinoma, adjuvant chemotherapy (SC and/or PIC) over 6 cycles and without SAE. These features could help better selecting patients from this category for such combined treatment in the future.

The differences in OS definition between our study and many other studies made it not sensible for direct comparison. In our study, the OS was defined as the time interval from the first surgery to death due to the disease for synchronous PC, and from CRS+HIPEC to death due to the disease for metachronous PC. Therefore, the objective of this definition was to evaluate the impact of CRS+HIPEC on survival. In our definition, the OS is the entire survival time for patients with synchronous PC. For patients with metachronous PC, the time interval from first surgery to the subsequent development of PC was not included in our OS consideration. In this study, the median OS was 22.2 months in synchronous subgroup and 14.5 months in metachronous subgroup, respectively (*P* = 0.09). Of course, the actual survival time in metachronous PC group could have been much longer if it had been estimated from the first treatment of colorectal cancer. In other studies, however, the definition was different. They defined OS as the time interval from first diagnosis to cancer-related death. For example, in a recent study by Kerscher et al [49] focusing on a comprehensive large CRC database (n = 2,406) analysis to decipher the characteristics of PC, the “OS was estimated from the diagnosis of the primary tumour”. Therefore, this study revealed the median OS was 8.0 months for patients with synchronous PC (n = 115), and 30.0 months for patients with metachronous PC (n = 141). Another example is by Jayne et al [50] studying 3,019 CRC patients, including 214 cases with synchronous PC and 135 cases with metachronous PC. Again, this study also defined OS as the time interval from “initial diagnosis of colorectal cancer”. The median OS was 7.0 months for synchronous PC and 28.0 months for metachronous PC (*P*<0.001). Therefore, because the definition of OS is different in our study, we just to emphasize that CRS+HIPEC could have better effects on selected patients with synchronous PC than those with metachronous PC. However, our multivariate analysis demonstrated that synchronous/metachronous PC was not an independent prognostic factor.

The OS differences between our study and those in the West could also be accounted for by the tumor biological differences between the Chinese and Western colorectal cancer patients. It has already been documented that Chinese colorectal cancer patients are approximately 10 years younger than those in the West [51], and tumor biology in younger patients are often more aggressive and lethal. Another fact is significantly higher percentages of rectal cancer origin in Chinese colorectal cancer patients. Table S5 summarized 9 representative studies to illustrate such differences. Among the 7 studies from China, including 6 from mainland China [52]–[57] and 1 from Hong Kong [58] on Chinese residents, over 40% of colorectal cancer patients were rectal origin. In comparison, among the 2 Western studies including one from UK [59] and another from France [16], less than 30% of patients were rectal primaries. Therefore, these differences could have considerable impacts on tumor biology, treatment strategy and clinical outcomes.

## Conclusion

In summary, this phase II study has provided additional evidence from China that CRS+HIPEC could bring survival benefits for selected CRC PC patients at specialized treatment centers.

## Supporting Information

Table S1 **Intraoperative parameters.**
(DOC)

Table S2 **OS comparisons stratified by major clinico-pathological factors.**
(DOC)

Table S3 **Analysis of independent factors influencing survival for patients with HPCI.**
(DOC)

Table S4 **Major studies on CRS+HIPEC, either single-arm or controlled studies.**
(DOC)

Table S5 **Nine large clinical-demographic studies on Chinese and Western colorectal cancer patients.**
(DOC)

Data S1 **RAW data for manuscript PONE-D-14-23603.**
(XLSX)
