# Supplementary material for: Cytoreductive Surgery plus Hyperthermic Intraperitoneal Chemotherapy Improves Survival for Patients with Peritoneal Carcinomatosis from Colorectal Cancer: A Phase II Study from a Chinese Center
Source: PLoS One. 2014 Sep 26;9(9):e108509. doi: 10.1371/journal.pone.0108509 (PMC4178169; doi:10.1371/journal.pone.0108509)
Supplement: Table S1 — Intraoperative parameters. (DOC) [file pone.0108509.s001.doc]

| **Table S1.** Intraoperative parameters ^a^ | | |
| --- | --- | --- |
| Items | Median | Range |
| Fluid output volume | | |
| Blood loss (mL) | 600 | 200 – 3,000 |
| Urine output (mL) | 1,000 | 200 – 3,000 |
| Ascites (mL) | 400 | 0 – 4,000 |
| Fluid intake volume | | |
| Plasma (mL) | 400 | 200 – 1,350 |
| RBC (u) ^b^ | 2.5 | 0 – 10 |
| Cryoprecipitation (u) ^c^ | 4 | 0 – 13 |
| Other fluids (mL) ^d^ | 4,000 | 300 – 7,500 |
| Duration of anesthesia (min) | 530 | 240 – 780 |
| Adjusted CRS time (excluding the HIPEC) (min) | 390 | 120 – 650 |
| ^a^ Values are in median (range), and a total of 63 procedures were performed for 60 patients, | | |
| including 3 patients each underwent 2 operations; | | |
| ^b^ 1 u = 200 mL; | | |
| ^c^ 1 u = 25 mL; | | |
| ^d^ Including colloids and electrolytes solution. | | |
