# Supplementary material for: Cytoreductive Surgery plus Hyperthermic Intraperitoneal Chemotherapy Improves Survival for Patients with Peritoneal Carcinomatosis from Colorectal Cancer: A Phase II Study from a Chinese Center
Source: PLoS One. 2014 Sep 26;9(9):e108509. doi: 10.1371/journal.pone.0108509 (PMC4178169; doi:10.1371/journal.pone.0108509)
Supplement: Table S2 — OS comparisons stratified by major clinico-pathological factors. (DOC) [file pone.0108509.s002.doc]

| **Table S2.** OS comparisons stratified by major clinico-pathological factors ^*^ | | | | | | | | | | | | |
| --- | --- | --- | --- | --- | --- | --- | --- | --- | --- | --- | --- | --- |
| Variables | n | | | | Median OS (mo) | | | | | | 95% CI (mo) | *P* |
| Gender | | | | | | | | | | | | 0.23 |
| Male | 26 | | | 22.1 | | | | | | 11.2 – 33.0 | |  |
| Female | 34 | | | 13.0 | | | | | | 9.2 – 16.6 | |  |
| Age (yr) | | | | | | | | | | | | 0.17 |
| < 60 | 46 | | 15.0 | | | | | 12.3 – 17.7 | | | |  |
| ≥ 60 | 14 | | Not reached | | | | | Not reached | | | |  |
| Primary tumor | | | | | | | | | | | | 0.09 |
| Carcinoma of colon | 35 | | | 22.2 | | | | | 10.5 – 33.9 | | |  |
| Carcinoma of rectum | 25 | | | 15.0 | | | | | 12.6 – 17.4 | | |  |
| Histopathology | | | | | | | | | | | | 0.12 |
| Adenocarcinoma, | 26 | | | Not reached | | | | Not reached | | | |  |
| well/intermediately differentiated |
| Adenocarcinoma, poorly/ | 34 | | | 15.0 | | | | 13.4 – 16.6 | | | |  |
| mucinous/signet-ring cell carcinoma |
| PC timing | | | | | | | | | | | | 0.09 |
| Synchronous | 24 | | | 22.2 | | | | | 11.1 – 33.3 | | |  |
| Metachronous | 36 | | | 14.5 | | | | | 10.1 – 18.9 | | |  |
| PCI scores | | | | | | | | | | | | **0.04** |
| ≤ 20 | 28 | 21.7 | | | | | | 13.0 – 30.4 | | | |  |
| > 20 | 32 | 14.8 | | | | | | 13.0 – 16.6 | | | |  |
| CC scores | | | | | | | | | | | | **0.01** |
| 0-1 | 32 | | 22.1 | | | | Not reached | | | | |  |
| 2-3 | 28 | | 14.5 | | | | 11.9 – 17.1 | | | | |  |
| Postoperative chemotherapy cycles | | | | | | | | | | | | **< 0.001** |
| < 6 | 15 | | | 8.5 | | 6.4 – 10.6 | | | | | |  |
| ≥ 6 | 45 | | | 22.1 | | 16.3 – 27.9 | | | | | |  |
| SAE | | | | | | | | | | | | 0.99 |
| No | 45 | | 16.0 | | | | 12.2 – 19.8 | | | | |  |
| Yes | 15 | | 22.2 | | | | 11.7 – 32.7 | | | | |  |
| Ascites |  | |  | | | |  | | | | | 0.26 |
| ≤ 1,000 mL | 42 | | 18.0 | | | | 10.6 – 25.4 | | | | |  |
| > 1,000 mL | 18 | | 13.0 | | | | 8.9 – 17.1 | | | | |  |
| ^*^ In the original surgery calculation. | | | | | | | | | | | | |
| OS = overall survival, mo = months. | | | | | | | | | | | | |
