# Supplementary material for: Cytoreductive Surgery plus Hyperthermic Intraperitoneal Chemotherapy Improves Survival for Patients with Peritoneal Carcinomatosis from Colorectal Cancer: A Phase II Study from a Chinese Center
Source: PLoS One. 2014 Sep 26;9(9):e108509. doi: 10.1371/journal.pone.0108509 (PMC4178169; doi:10.1371/journal.pone.0108509)
Supplement: Table S3 — Analysis of independent factors influencing survival for patients with HPCI. (DOC) [file pone.0108509.s003.doc]

| **Table S3.** Analysis of independent factors influencing survival for patients with HPCI | | | | | | | | | |
| --- | --- | --- | --- | --- | --- | --- | --- | --- | --- |
| Covariate | Survival analysis | | | |  | Multivariate analysis | | | |
|  | n | Median OS, mo | 95% CI, mo | *P* |  | χ^2^ | HR | 95% CI | *P* |
| PC timing |  |  |  | 0.12 |  | 3.34 | 2.9 | 0.9 – 8.9 | 0.68 |
| Syn | 15 | 15.0 | 13.0 – 17.0 |  |  |  |  |  |  |
| Met | 17 | 9.5 | 2.0 – 17.0 |  |  |  |  |  |  |
| PCC |  |  |  | **< 0.001** |  | 14.26 | **15.0** | 3.7 – 61.0 | **< 0.001** |
| < 6 | 9 | 6.0 | 5.4 – 6.6 |  |  |  |  |  |  |
| ≥ 6 | 23 | 17.8 | 10.8 – 24.8 |  |  |  |  |  |  |
| CC score |  |  |  | 0.16 |  | 1.05 | 3.0 | 0.4 – 23.3 | 0.30 |
| 0-1 | 7 | not reached | not reached |  |  |  |  |  |  |
| 2-3 | 25 | 14.5 | 12.1 – 16.9 |  |  |  |  |  |  |
| SAE |  |  |  | 0.21 |  | 0.82 | 1.8 | 0.5 – 6.0 | 0.37 |
| No | 23 | 14.8 | 11.0 – 18.6 |  |  |  |  |  |  |
| Yes | 9 | 13.7 | 2.6 – 24.8 |  |  |  |  |  |  |
| PCC = postoperative chemotherapy cycles, Syn = synchronous, Met = metachronous, mo = months. | | | | | | | | | |
