# Supplementary material for: Cytoreductive Surgery plus Hyperthermic Intraperitoneal Chemotherapy Improves Survival for Patients with Peritoneal Carcinomatosis from Colorectal Cancer: A Phase II Study from a Chinese Center
Source: PLoS One. 2014 Sep 26;9(9):e108509. doi: 10.1371/journal.pone.0108509 (PMC4178169; doi:10.1371/journal.pone.0108509)
Supplement: Table S4 — Major studies on CRS+HIPEC, either single-arm or controlled studies. (DOC) [file pone.0108509.s004.doc]

| **Table S4.** Major studies on CRS+HIPEC, either single-arm or controlled studies. | | | | | | | | | | | | | | |
| --- | --- | --- | --- | --- | --- | --- | --- | --- | --- | --- | --- | --- | --- | --- |
| Author/Years  /Country/Level | n | CRS+HIPEC  (n or %) | HIPEC procedure | Median OS of CC (n or %, mo) | SC/EPIC | Median OS of PCI (n or %, mo) | Median OS (n or %, mo) | PFS  (mo) | Morbidity | Mortality | 1-yr OS rate | 2-yr OS rate | 3-yr OS rate | 5-yr OS rate |
| Witkamp et al.^20^ 2001, Holland phase I | 29 | 29 | inflow 43℃, stable T 40℃, MMC (35 mg/m^2^) added in 3 doses at 30 min interval, 50%, 25%, 25% of the total dose. 90 min. open technique | CC0/1：26/29 | SC: 5-FU (400 mg/m^2^) + CF (80 mg/m^2^)  21/29 | NR | NR | NR | 37.9 % | 3.4 % | 82.0 %; | 45.0 % | 23.0 % | NR |
| Glehen et al.^25^ 2003, France, phase II | 56 | 56  CRC: 26 | MMC (0.7 mg/kg, maximum dose of 60 mg) for 90 min at 46-48℃ open technique | CC0/1：27/56 | EPIC: MMC (0.7 mg/kg, maximal 60 mg) | NR | NR | NR | 16/56 (28.6 %) | 1/56 (1.8 %) | NR | CC0: 79.0 %  CC1-2: 44.7 % | NR | NR |
| Verwaal.et al.^6^ 2003, Holland, phase III | 105 | 54 | MMC stable at 41 – 42 ℃, at first added to17.5 mg/m^2^ followed by 8.8 mg/m^2^ every 30 minutes; for 90 min. open technique | CC0: 18/49 (36.7 %)  CC1: 21/49 (42.9 %) | SC: 5-FU (400 mg/m^2^) + CF (80 mg/m^2^)  Control: 44/51  Study: 33/54 | NR | Control : 12.6  Study: 22.2 | Control: 7.7  Study: 12.6 | Control: 2/51 (3.9 %) | Study: 4/48 (8.3%) | CC0: 95.0%  CC1: 66.0%  CC2-3:21.0% | CC0: 75.0%  CC1: 20.0%  CC2-3:11.0% | CC0: 57.0%  CC1: 10.0% | CC0: 45.0 %  CC1: 5.0 % |
| Rouers et al.^26^ 2006, Belgium, phase II | 21 | 21 | 13 patients with MMC (10 mg/m^2^) and 8 patients with OX (460 mg/m^2^) for 90 min at 41-42.5 ℃; 1h before HIPEC iv 5-FU (400 mg/m^2^) + CF (20 mg/m^2^) in all patients.  open technique | 21/21 (100 %) | NR | PCI < 15: 8.28 | 34.0 | NR | 33.3 % | 7.7 % (1/13) in MMC group | MMC group: 88.7 %  OX group: 72.6 % | MMC group  72.9 %  OX group: 37.1 % | MMC group:  45.5 %  OX group: 36.6 % | Overall: 36.6 % |
| Elias et al.^27^ 2007, France, phase II | 106 | 106 | inflow 43℃ (42-45℃), OX+IR (460 mg/m^2^) for 30min at ; 1 hour before HIPEC, 5-FU (400 mg/m^2^) + CF (20 mg/m^2^) administered  systemic IV  open technique | 106/106 (100 %) | NR | NR | NR | NR | 66.0 % | 4.0 % | NR | NR | NR | NR |
| Stewart et al.^21^ 2008, American phase I | 15 | 15  CRC: 3  APC: 2 | OX (200 and 250 mg/m^2^) for 120 min at 40℃ (39-42.5 ℃)  open technique | 9/15 (60.0 %) | NR | NR | NR | NR | NR | NR | NR | NR | NR | NR |
| Harrison et al.^22^ 2008, American phase I | 21 | 21  CRC: 9 | Pegylated liposomal doxorubicin (PLD) (15-100 mg/m^2^) for 90 min at 40℃  open technique | 12/21 (57.1 %) | NR | NR | all tumor: 30.6 | 25 all tumor PFS | 9/21 (42.9 %) | 0 | NR | NR | NR | NR |
| Ceelen et al.^23^ 2008, Belgium, phase I | 52 | 32  CRC: 32 | OX (460 mg/m^2^) for 30 min at 41-42℃  open technique | 60.0 % | NR | NR | 14.5 mo all tumor  CRC: not reached | NR | 24.0% | 0 | 80.0% | NR | NR | NR |
| van Leeuwen et al.^28^ 2008, Sweden, phase II | 103 | 38  CRC: 38 | OX (460 mg/m^2^) for 30 min at 42-44℃ ; concomitant 5-FU (500 mg/m^2^) + isovorin (30 mg/m^2^) IV  open technique | NR | EPIC: 5-FU (550 mg/m^2^)  Postoperative days 1-5 daily  SC: FOLFOX in 2 patients | NR | About 14 | NR | NR | NR | 82.0% | 63.7% | 63.7% | NR |
| Yan TD, et al.^29^2008 Australia, phase II | 50 | 50 | MMC (10–12.5 mg/m^2^) for 90 min at 42℃;  open technique | 41/50 (82.0 %) | EPIC: 5-FU (650–800 mg/m^2^) on POD 1–5 per day  SC regimen unclear | ≥ 10 and < 20: 29  ≥ 20: 27 | Overall: 29.0  CC0: 37  CC1-3: 14 | NR | 76.0 % | 0 | 79.0% | 67.0% | 39.0% | NR |
| Sideris et al.^30^ 2009, France, phase II | 37 | APC: 11 | OX (460 mg/m^2^)for 35 min at 42-43℃  open technique | NR | SC: 5-FU+ irinotecan or OX | NR | NR | NR | 36.0% | 0 | 90.0% | 65.0% | NR | 60.0 % |
| Cotte et al.^24^ 2011, France, phase I | 12 | 12  CRC: 1 | MMC (0.7 mg/kg) for 90 min and irinotecan (100 mg/m^2^ and 150 mg/m^2^ , respectively) added at the final 30 min inflow temperatures 44-46℃  open technique | NR | NR | NR | NR | NR | NR | NR | NR | NR | NR | NR |
| Quenet et al.^31^ 2011, France, phase II | 146 | 146 | OX-alone group (460 mg/m^2^) and OX-irinotecan group (OX, 460 mg/m^2^; irinotecan, 200 mg/m^2^) for 30 min at 42-45 ℃  open technique  before HIPEC, IV 5-FU (400 mg/m^2^)+ CF (20 mg/m^2^) | Overall: 132/146 (90.4 %)  OX-alone: 32/43 (74.4 %)  OX-irinotecan: 100/103 (97.1 %) | NR | NR | Overall: 41  OX-alone: 40.8  OX-irinotecan: 47 | Overall: 15.7  OX-alone: 16.8  OX-irinotecan: 15.7 | Overall: 47.2%  OX-alone: 34.9%  OX-irinotecan: 52.4% | Overall: 4.1% | NR | NR | NR | OX-alone: 41.8 %  OX-irinotecan: 42.4 %  PCI <10: 26.0%  PCI 11-19: 26.0%  PCI ≥20: 18.0% |
| Hompes et al.^32^ 2012, Belgium, phase II | 48 | 48 | OX 460 mg/m^2^ for 30 min at 41-42 ℃  open technique  1 hour before HIPEC, IV 5-FU (400 mg/m^2^)+ CF (20 mg/m^2^). | 48/48 (100 %) | 5-FU/CF+OX or irinotecan within 8–12 weeks after CRS + HIPEC | NR | 19.8 | 1-yr: 65.8 %  2-yr: 45.5 % | 52.1 % | 0 | 97.9% | 88.7 % | NR | NR |
| Glockzin et al.^33^ 2013, Germany, phase II | 60 | 60 | inflow 40^0^C, OX (300 mg/m^2^) at 30 min and synchronous IV 5-FU (400 mg/m^2^) + CF (20 mg/m^2^)  open technique | 60/60 (100 %) | Preoperative and postoperative chemotherapy, 3 months  FOLFOX/FOLFIRI + Cetuximab (initial dose 400 mg/m^2^ and weekly dose 250 mg/m^2^), respectively | NR | NR | NR | NR | NR | NR | NR | NR | NR |
| PFS = progression free survival, OX = oxaliplatin, CF = calcium foliate, APC = appendix mucinous carcinoma, CRC = colorectal carcinoma, OS = overall survival, mo = months, yr = year, NR = not reported, CC = completeness of | | | | | | | | | | | | | | |
| cytoreduction, SC = systemic chemotherapy, EPIC = early postoperative intraperitoneal chemotherapy. | | | | | | | | | | | | | | |
