# Supplementary material for: Cytoreductive Surgery plus Hyperthermic Intraperitoneal Chemotherapy Improves Survival for Patients with Peritoneal Carcinomatosis from Colorectal Cancer: A Phase II Study from a Chinese Center
Source: PLoS One. 2014 Sep 26;9(9):e108509. doi: 10.1371/journal.pone.0108509 (PMC4178169; doi:10.1371/journal.pone.0108509)
Supplement: Table S5 — Nine large clinical-demographic studies on Chinese and Western colorectal cancer patients. (DOC) [file pone.0108509.s005.doc]

| **Table S5.** Nine large clinical-demographic studies on Chinese and Western colorectal cancer patients. | | | | | |
| --- | --- | --- | --- | --- | --- |
| References | Location of tumor (n/%) | | Histology (n/%) | | Total (n) |
|  | Rectum | Colon | Well/Moderately-differentiated | Poorly-differentiated |  |
| ***Chinese studies*** | | | | | |
| Zheng et al.^52^ | 420 (57.7) | 308 (42.3) | NR | NR | 728 |
| Fu et al.^53^ | 662 (49.6) | 673 (50.4) | 922 (69.1) | 413 (30.9) | 1,335 |
| Xu et al.^54^ | 112 (50.5) | 110 (49.5) | 194 (87.4) | 28 (12.6) | 222 |
| Xu et al.^55^ | 4,434 (54.3) | 3,738 (45.7) | 4,920/6,638 (74.8) | 1,718/6,638 (25.2) | 8,172 |
| Zhang et al.^56^ | 1,467 (51.0) | 1,396 (48.5) | 371/1,918 (19.3) | 1,547/1,918 (80.7) | 2,863 |
| Wang et al.^57^ | 155 (47.5) | 171 (52.5) | 275 (84.4) | 51 (15.6) | 326 |
| Ho et al.^58^ | 357 (44.1) | 452 (55.9) | NR | NR | 809 |
| ***Western studies*** | | | | | |
| Logan et al.^59^ | 496 (28.7) | 1,234 (71.3) | NR | NR | 1,730 |
| Elias et al.^16^ | 36 (6.9) | 414 (79.1) | 360/425 (84.7) | 65/425 (15.3) | 523 |
| NR = not reported. | | | | | |
